# Chronic Exposure to Endocrine Disruptor Vinclozolin Leads to Lung Damage via Nrf2–Nf-kb Pathway Alterations

**DOI:** 10.3390/ijms231911320

**Published:** 2022-09-26

**Authors:** Ramona D’Amico, Davide Di Paola, Daniela Impellizzeri, Tiziana Genovese, Roberta Fusco, Alessio Filippo Peritore, Enrico Gugliandolo, Rosalia Crupi, Livia Interdonato, Salvatore Cuzzocrea, Rosanna Di Paola, Rosalba Siracusa, Marika Cordaro

**Affiliations:** 1 Department of Chemical, Biological, Pharmaceutical and Environmental Sciences, University of Messina, Viale Ferdinando Stagno D’Alcontres 31, 98166 Messina, Italy; 2 Department of Veterinary Sciences, University of Messina, 98168 Messina, Italy; 3 Department of Pharmacological and Physiological Science, Saint Louis University School of Medicine, Saint Louis, MO 63104, USA; 4 Department of Biomedical, Dental and Morphological and Functional Imaging, University of Messina, Via Consolare Valeria, 98125 Messina, Italy

**Keywords:** endocrine disruptor, lung injury, inflammation, oxidative stress, apoptosis, vinclozolin

## Abstract

Endocrine-disrupting substances (EDS) are common and pervasive in our environment and pose a serious risk to both human and animal health. Endocrine-disrupting compounds (EDCs) have been associated with a variety of detrimental human health effects, including respiratory issues, as a result of their ability to disrupt cell physiology. Vinclozolin ((RS)-3-(3,5-Dichlorophenyl)-5-methyl-5-vinyloxazolidine-2,4-dione) is a common dicarboximide fungicide used to treat plant diseases. Several studies have analyzed the effects of vinclozolin exposure on the reproductive system, but less is known about its effect on other organs such as the lung. Mice were exposed for 28 days to orally administered vinclozolin at a dose of 100 mg/kg. Vinclozolin exposure induced histological alterations and collagen depositions in the lung. Additionally, vinclozolin induced inflammation and oxidative stress that led to lung apoptosis. Our study demonstrates for the first time that the toxicological effects of vinclozolin are not limited to the reproductive system but also involve other organs such as the lung.

## 1. Introduction

In vineyards and on fruits and vegetables such as raspberries, lettuce, kiwifruit, snap beans, and onions, vinclozolin ((RS)-3-(3,5-Dichlorophenyl)-5-methyl-5-vinyloxazolidine-2,4-dione), a popular dicarboximide fungicide, is used to treat diseases such as blights, rots, and molds. Additionally, it is applied to golf course turf. Vinclozolin, which was first registered in 1981, is widely utilized, but its total application has decreased. Vinclozolin is subject to Environmental Protection Agency (EPA) regulation as a pesticide in the United States (U.S. EPA) [1,2]. In addition to these restrictions inside the United States, as of 2006, the use of this pesticide was restricted in numerous nations, including Denmark, Finland, Norway, and Sweden. To assess its risks and hazards to the environment and animals, a number of tests and regulations have been applied [3,4]. Vinclozolin is created as extruded or dried flowable granules. Aerial application, chemigation through irrigation systems, and ground machinery are all options for distribution. The U.S. EPA has investigated occupational exposure to vinclozolin or its metabolites, as well as dietary (food and water) and non-dietary exposure. Fungicides can wind up on untreated foods following application because it has been demonstrated that they circulate through the water and air. Because fungicides are not eliminated from produce when it is washed with tap water, consumers cannot easily minimize their exposure [5]. One significant way that vinclozolin can be ingested is through wine grapes, which are thought to account for around 2% of all vinclozolin exposure. Tolerance limits have been defined for each crop because it has been determined that people may be exposed to vinclozolin residues and metabolites that contain the 3,5-dichloroaniline moiety (3,5-DCA) through eating. It is nevertheless feasible for humans to encounter vinclozolin and its residues, even if it is not approved for use by homeowners. Golfers playing on treated courses and families playing on previously treated sod, for instance, may be at risk for exposure. Vinclozolin exposure might occur at work when performing tasks such as loading and mixing. Vinclozolin has been demonstrated to be an endocrine disruptor with antiandrogenic effects, which is one of the major research findings [6,7]. Moreover, less is known about the effects of vinclozolin exposure on human health in other organs. Gazo and colleagues demonstrated that one of the mechanisms by which vinclozolin induces sperm alterations is an increase in oxidative stress in the testis [8]. In particular, they demonstrated that an increase in reactive oxygen species (ROS) production, as well as in lipid peroxidation, led to DNA damage. This was reflected in altered sperm mobility and velocity [8]. Additionally, they demonstrated an alteration in antioxidant endogenous defense, reflected in an increase in Mn-SOD activity after vinclozolin exposure [8]. Following this study, D’Amico et al. demonstrated that exposure to vinclozolin (and other endocrine disruptors) significantly induced oxidative stress and inflammation, worsening arthritis and Parkinson’s disease in experimental murine models [9,10]. In consideration of the fact that most previous studies investigated the effects of vinclozolin exposure on the reproductive system, we investigated for the first time the impact of long exposure to vinclozolin on the respiratory system [11,12].

## 2. Results

### 2.1. Exposure to Vinclozolin Induced Lung Alterations

**Figure 1:**
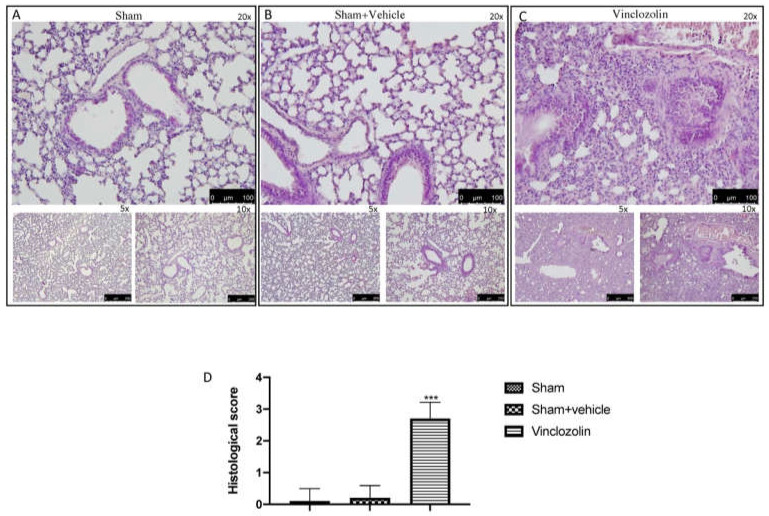
Vinclozolin-induced histological alterations. Square (**A**): images of sham animals at different magnifications (5×, 10×, and 20×); Square (**B**): images of sham + vehicle animals treated for 28 days with corn oil at different magnifications (5×, 10×, and 20×); Square (**C**): images of vinclozolin-treated animals at different magnifications (5×, 10×, and 20×); (**D**): Histological score. After vinclozolin administration, we observed a moderate thickening of the walls without obvious damage to the lung architecture. Values are the means ± SD of 6 mice per group. Images shown are representative of the results obtained. See manuscript for further details. *** p < 0.001 vs. sham.

We decided to explore the histological damage to the lungs due to exposure to vinclozolin. Figure 1 presents a comparison of the microarchitecture of the lungs among the experimental groups of the control and vinclozolin-treated animals at different magnifications (5×, 10×, and 20×). We found no signs of inflammation in either control group (Figure 1 square A for sham animals and square B for animals treated with corn oil, with histological score D). On the other hand, vinclozolin treatment induced significant inflammatory infiltrate in the lungs, with an increase in the alveolar wall and slight congestion with slight exudation in the alveolar spaces and lung interstitium, as judged from the 20× magnification in the center of the image (Figure 1 square C and histological score D).

### 2.2. Exposure to Vinclozolin Induced Lung Fibrosis

**Figure 2:**
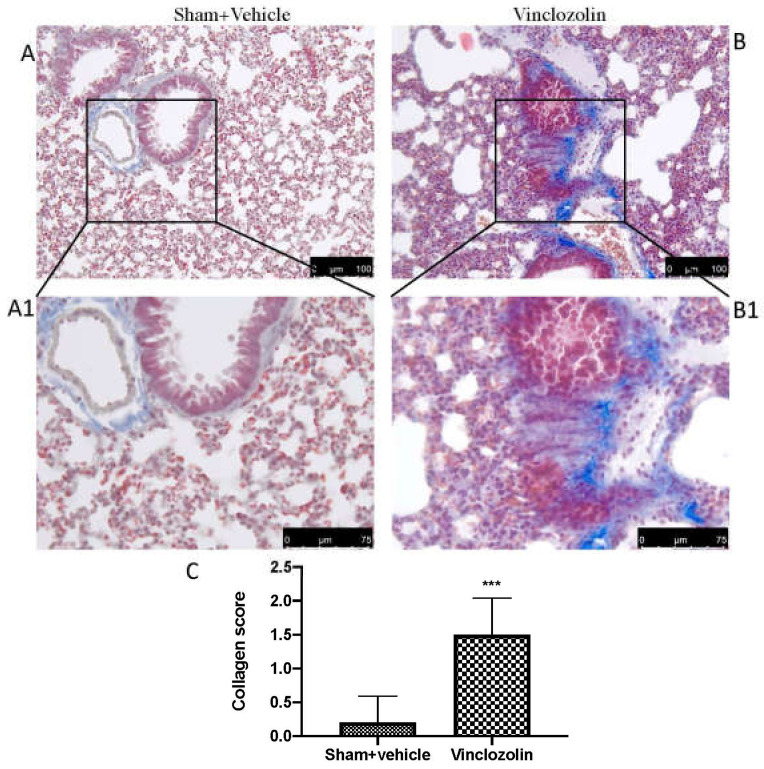
Collagen deposition after exposure to vinclozolin. Masson trichrome staining results for sham (**A**,**A1**) and for vinclozolin-treated (**B**,**B1**) animals; collagen score (**C**). Values are the means ± SD of 6 mice per group. Images shown are representative of the results obtained. After vinclozolin exposure, we found a significant increase in collagen deposition as shown by the increase in blue staining. *** p < 0.001 vs. sham.

To assess pulmonary fibrosis in lung tissue, Masson’s trichrome staining was used. When compared to the sham group, vinclozolin administration caused fibrotic lesions in which the alveolar walls presented an increased amount of blue-stained fibrous connective tissue (Figure 2B, greater magnification B1). On the other hand, only a physiological deposition of collagen was seen in the sham group (Figure 2A, greater magnification A1).

### 2.3. Exposure to Vinclozolin Induced Inflammation and Oxidative Stress

**Figure 3:**
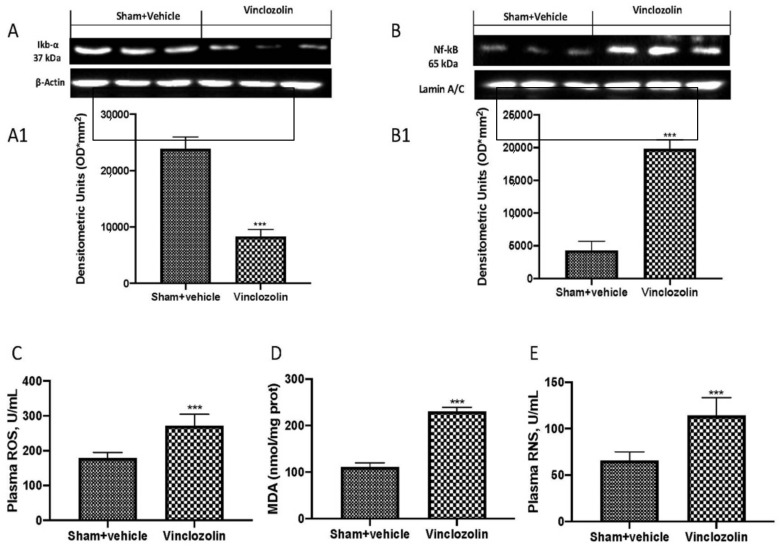
Exposure to vinclozolin induces Nf-kb activation and oxidative stress. Western blots and respective quantification of Ikb-α (**A**,**A1**) and Nf-kb pathway (**B**,**B1**); ELISA kit results in plasma for ROS (**C**), H_2_O_2_ (**D**), and reactive nitrogen species (RNS) (**E**). As shown, vinclozolin exposure increased inflammation and oxidative stress. Values are the means ± SD of 6 mice per group. *** *p* < 0.001 vs. sham.

To investigate the molecular mechanism by which vinclozolin induces lung alterations, we performed Western blots of the Nf-kb pathway. We found a significant increase in Ikb-α degradation (Figure 3A and densitometric analysis A1), as well as an increase in Nf-kb nuclear translocation (Figure 3B and densitometric analysis B1) after vinclozolin exposure.

The increased generation of reactive oxygen species suggests that oxidative stress may play a role in vinclozolin-induced toxicity. For this reason, we investigated, by ELISA kits, the reactive oxygen species (ROS) production (Figure 3C), as well as H_2_O_2_ (Figure 3D) and reactive nitrogen species (RNS) (Figure 3E). We found that vinclozolin induced a significant increase in all these parameters.

**Figure 4:**
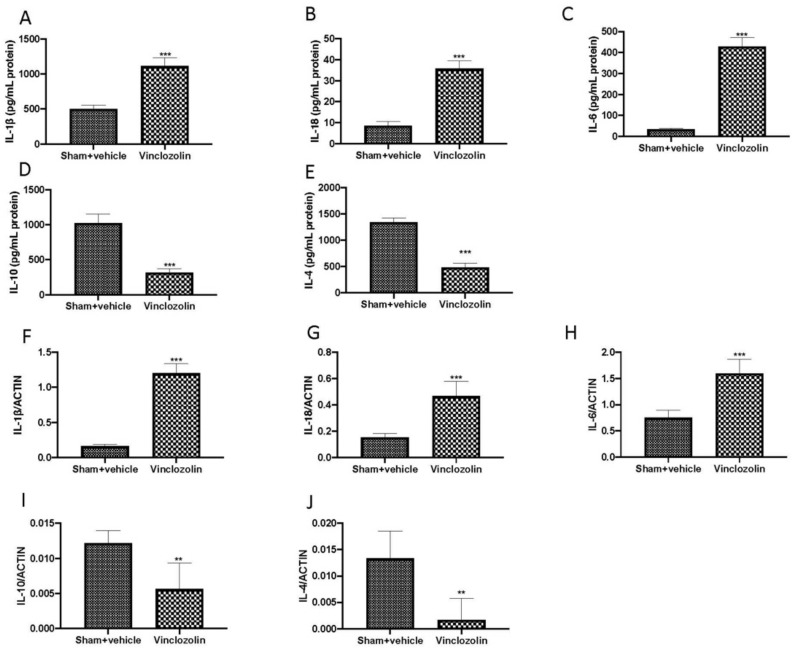
Cytokine analysis after vinclozolin exposure. ELISA kit results for IL-1β (**A**), IL-18 (**B**), IL-6 (**C**), IL-10 (**D**), and IL-4 (**E**). RT qPCR results for IL-1β (**F**), IL-18 (**G**), IL-6 (**H**), IL-10 (**I**), and IL-4 (**J**). As shown, after vinclozolin exposure, there was a significant increase in proinflammatory cytokines, as well as a significant decrease in anti-inflammatory cytokines. Values are the means ± SEM of 6 mice for all groups. ** *p* < 0.01 vs. sham, *** *p* < 0.001 vs. sham.

Additionally, numerous interleukins (ILs), which cause problems with cellular communication, are released when endocrine disruptors (EDs) are present, according to numerous independent investigations [13]. Using ELISA kits, we discovered that vinclozolin exposure increased the release of pro-inflammatory cytokines in BALF, specifically, IL-1β (Figure 4A), IL-18 (Figure 4B), and IL-6 (Figure 4C), whereas we discovered considerably lower levels of anti-inflammatory cytokines IL-10 (Figure 4D) and IL-4 (Figure 4E) when compared to sham groups.

**Figure 5:**
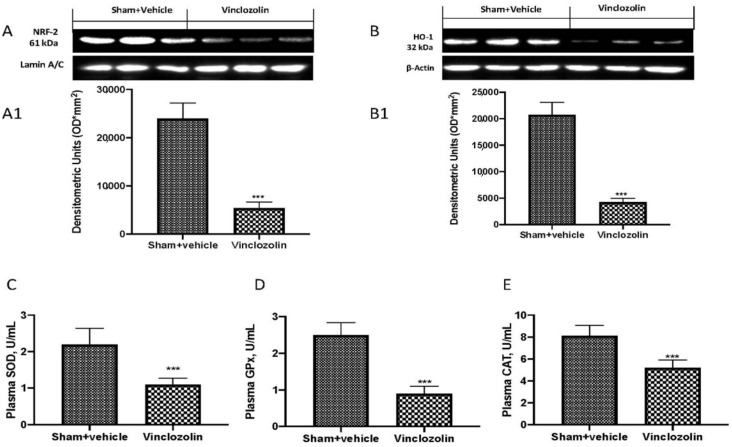
Vinclozolin-induced Nrf-2 pathway alteration. Western blots and quantification of NRF2 (**A**,**A1**) and HO-1 (**B**,**B1**) in lung tissue. ELISA kit results for SOD (**C**), GPx (**D**), and CAT (**E**) levels. Vinclozolin exposure induced a significant alteration in the Nrf-2 pathway with an imbalance in antioxidant defense. Values are the means ± SD of 6 mice per group. *** *p* < 0.001 vs. sham.

When exposed to oxidative injury, organisms have evolved defensive mechanisms that include Nrf-2-HO-1 pathway activation and antioxidant enzymes such as superoxide dismutase (SOD), glutathione peroxidase (GPx), and catalase (CAT). By Western blotting, we found a significant decrease in Nrf-2 (Figure 5A and densitometric analysis A1) and HO-1 (Figure 5B and densitometric analysis B1) and, as a consequence, a significant decrease in SOD (Figure 5C), GPx (Figure 5D), and CAT (Figure 5E).

### 2.4. Exposure to Vinclozolin Induced DNA Damage and Lipid Peroxidation

**Figure 6:**
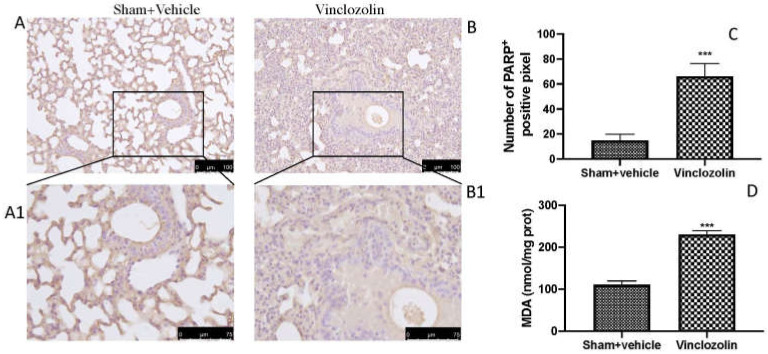
Exposure to vinclozolin induced DNA damage and lipid peroxidation. Immunohistochemistry localization of PARP for sham (**A**,**A1**) and for vinclozolin-treated (**B**,**B1**) animals; score (**C**); MDA levels (**D**). Vinclozolin induced an increase in PARP expression, as well as in lipid peroxidation. Values are the means ± SD of 6 mice per group. Images shown are representative of the results obtained. *** p < 0.001 vs. sham.

Exposure to endocrine disruptors has been linked to epigenetic alterations in a variety of tissues and organisms [14,15]. We investigated PARP by immunohistochemistry, and we found a significant increase in PARP-positive cells after vinclozolin exposure (Figure 6B with higher magnification B1 and analysis C) compared to sham (Figure 6A with higher magnification A1 and analysis C). Vinclozolin also significantly induced lipid peroxidation, as demonstrated by MDA analysis (Figure 6D).

### 2.5. Exposure to Vinclozolin Induced Lung Apoptosis

**Figure 7:**
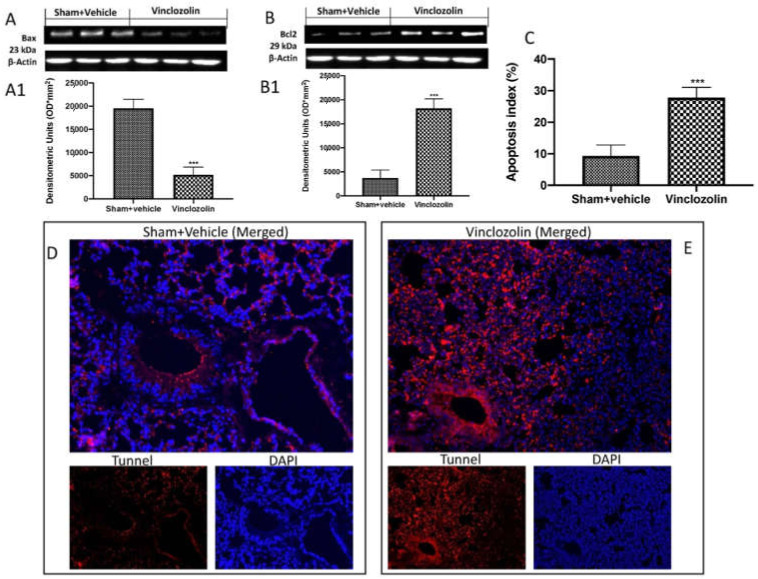
Vinclozolin exposure induced lung apoptosis. Western blots and quantification of lung tissue from Bax (**A**,**A1**) and Bcl-2 (**B**,**B1**); TUNEL images from lung tissue of sham animals (**D**) and vinclozolin-treated animals (**E**); apoptotic index (**C**). After 28 days of vinclozolin exposure, we found a significant increase in cell death. Values are the means ± SD of 6 mice per group. Images shown are representative of the results obtained. *** p < 0.001 vs. sham.

To further investigate the effects of vinclozolin exposure on the lung, we conducted a Western blot analysis of Bax and Bcl-2. We found that animals subject to vinclozolin exposure showed a significant decrease in Bax expression (Figure 7A and densitometric analysis A1) and, as a consequence, a significant increase in Bcl-2 expression (Figure 7B and densitometric analysis B1) compared to the control group. Our data were also confirmed by TUNEL analysis of lung sections. As shown in square D, sham animals showed physiological apoptosis. Vice versa, in square E, animals exposed to vinclozolin showed a significant increase in apoptotic cells (see apoptosis index C).

## 3. Discussion

Public interest in endocrine-disrupting chemicals’ impacts on health is growing, especially those related to long-term, low-dose exposures [16]. “Agent that interferes with the synthesis, secretion, transport, binding, or elimination of natural hormones in the body that are responsible for the maintenance of homeostasis, reproduction, development, and/or behavior” is how the U.S. Environmental Protection Agency (EPA) defines an endocrine disruptor (ED) [17]. This definition is obsolete because many animal experiments, clinical observations, and epidemiological studies have shown that endocrine disruptors can have an impact on different organs such as the reproductive system, the prostate, the breast, the liver, the thyroid, and the lungs, as well as metabolism [18]. Unfortunately, the fact that EDCs are a “shifting target” makes it difficult to comprehend how they relate to health issues. People and populations are exposed to constantly shifting patterns of chemical production and consumption. Additionally, rather than being released into the environment as separate compounds, they are frequently released as mixes. Although it is challenging to establish a direct causal link between human exposures to EDCs and disease states, findings from fundamental research and epidemiological studies demonstrate the importance of expanding screening for exposures and focusing on at-risk populations. An essential and successful strategy is disease prevention, in addition to advancing research in these fields [19]. Vinclozolin is a fungicide commonly used in fruits, vegetables, ornamental plants, and turf grasses. Vinclozolin exposure at work can happen through skin contact and dust inhalation. It may be consumed by the general population if produce they eat has been tainted with the fungicide. Vinclozolin studies have mainly focused on the reproductive system. It was demonstrated that exposure between the embryonic day and the postnatal day results in anomalies of the external genitalia in the neonatal stage, including a shortening of the anogenital distance and the retention of nipples in male pups. Due to a cleft phallus and hypospadias, male rats do not develop intromission or ejaculation during maturation. Additionally, males exhibit ectopic testicles, vaginal pouches, epididymal granulomas, accessory sex glands that are smaller or missing, and fewer cauda epididymal sperm. Additionally, vinclozolin prepubertal exposure in male rats inhibits pubertal maturation and slows the growth of the accessory sex gland and epididymis [20]. D’Amico and colleagues recently demonstrated that the effect of endocrine disruptor exposure is not limited to urogenital apparatus [9,10]. Here, we demonstrated for the first time that long-term exposure to vinclozolin induced significant damage to the respiratory system. Several cytokines, in particular, are generated by monocytes and macrophages, which attract neutrophils into lung tissues. This is important for host defense and promotes the onset of lung injury [21]. On the other hand, powerful anti-inflammatory cytokines such as IL-4 and IL-10 may be able to reduce the production of several inflammatory molecules [21]. Vinclozolin previously demonstrated the ability to induce histological alterations in the testis, prostate, ovary, kidney, and gonadal fat pad [22]. In line with this research, we found that a daily oral administration of vinclozolin significantly induced histological alterations, as well as an increase in collagen deposition that led to lung fibrosis. Unfortunately, it is well known that endocrine disruptors play a key role in inflammation and oxidative stress. In fact, they induce a significant alteration in the Nf-kb and Nrf-2 (nuclear factor erythroid 2–related factor 2) pathways, of which are master regulators of two of the most important cellular responses. During inflammation, a range of cells, including immune cells such as macrophages and lymphocytes, as well as endothelial cells and fibroblasts, produce cytokines that function as messengers between immune cells to control cell maturation, development, and responsiveness [23,24]. Additionally, in a system where oxidant species predominate, oxidative stress is defined as an imbalance between oxidant and antioxidant species [25]. As a potential modulator of ED-related outcomes, it has only recently been examined [26]. Numerous techniques have been employed to measure oxidative stress, each with its own uses and interpretations. These techniques are relatively easy to apply in both animal and human investigations because several oxidative stress assessments may be obtained by minimally invasive techniques, for instance, in blood [27]. In our study, we found a significant increase in both the Nf-kb and Nrf-2 pathways, as well as an increase in pro-inflammatory cytokines and ROS production. Radice and colleagues demonstrated that vinclozolin was able to induce lipid peroxidation in the Hep92 cell line [28]. As previously proven in other cell types, we found a significant increase in DNA damage, verified by the activation of PARP, a well-known marker of DNA damage, as well as an increase in lipid peroxidation, proven via an MDA level investigation [29,30,31,32,33,34]. Different studies demonstrated that vinclozolin administration induces granulosa cell apoptosis during follicular atresia in pigs, as well as apoptosis in testis [35,36]. Here, we demonstrated that long-term exposure to vinclozolin significantly induced lung apoptosis, as confirmed by the decrease in Bax activation and increase in Bcl-2 expression and TUNEL signal.

## 4. Materials and Methods

### 4.1. Animals

CD1 male mice (8 weeks old, 18–24 g) were acquired from Envigo (Milan, Italy) and kept in a controlled environment. All animal experiments complied with the new Italian regulations (D. Lgs 2014/26), EU regulations (EU Directive 2010/63), and ARRIVE guidelines.

### 4.2. Experimental Design and Groups

Mice were arbitrarily divided into three groups:

- Sham: animals that were untreated (shown only in the histology);
- Sham+vehicle: animals that were orally administered with corn oil;
- Vinclozolin: animals that were orally administered 100 mg of vinclozolin daily for 28 days.

At the end of experiment, mice were sacrificed, and lung tissue and blood samples were collected for histology and biochemical analysis as previously described [37,38]. The dose, route of administration, and time of vinclozolin exposure were chosen based on another previous study and on a short exposure time at a different concentration observed in our laboratory (see Supplementary Figure S1) [9,10].

### 4.3. Western Blot Analysis of Cytosolic and Nuclear Extracts

Extracts from the cytosol and nucleus were prepared firstly with Buffer A containing 0.2 mM PMSF, 0·15 μM pepstatin A, 20 μM leupeptin, and 1 μM sodium orthovanadate, homogenized at the highest setting for 2 min, and centrifuged at 1000× *g* for 10 min at 4 °C. Following this, supernatants representing the cytosolic fraction and pellets containing enriched nuclei were re-suspended in Buffer B containing 1% Triton X-100, 150 mM NaCl, 10 mM Tris-HCl, pH 7.4, 1 mM EGTA, 1 mM EDTA, 0.2 mM PMSF, 20 μM leupeptin, and 0.2 mM sodium orthovanadate, as previously mentioned [39,40]. The following primary antibodies were used: anti-Iκbα (1:500, Santa Cruz Biotechnology (SCB), #sc-1643, Dallas, TX, USA), anti-nfκb (1:500, SCB, #sc8414), anti-NRF-2 (1-500, SCB, Heidelberg, Germany, #sc-365949), anti-heme oxygenase 1 (HO-1; 1-500, SCB, Heidelberg, Germany, #sc-136960), anti-Bax (1:500, SCB, #sc7480), and anti-Bcl-2 (1:500, SCB, #sc7382) in 1× PBS, 5% w/v non-fat dried milk, and 0.1% Tween-20 at 4 °C overnight. For the cytosolic and nuclear fractions, blots were also probed with β-actin and lamin A/C protein to ensure that they were filled with equivalent amounts of proteins (1:500; Santa Cruz Biotechnology). Signals were detected as previously described in our studies [40,41,42,43,44,45,46,47,48].

### 4.4. Histopathological Evaluation

Lungs were stained with hematoxylin/eosin (H/E) or Masson Trichrome and analyzed using a LeicaDM6 light microscope connected to an imaging system (LasX Navigator); they were blindly scored by two investigators without knowledge of the experimental groups. The severity of lung damage and fibrosis was assessed according to the method of Ashcroft et al. [29,49]. Briefly, histological alteration was quantified as follows: 0 for normal lung; 1 for minimal fibrous thickening of alveolar or bronchiolar walls; 3 for moderate thickening of the walls without obvious damage to lung architecture; 5 for increased fibrosis with definite damage to lung structure and the formation of fibrous bands or small fibrous masses; 7 for severe distortion of lung structure and large fibrous areas; 8 for total fibrous obliteration of fields. Collagen deposition was quantified as follows: 0 for negligible collagen deposition at the alveolar septa and thin-walled alveoli; 1 for one-layer deposition of collagen in the alveolar septa with mild fibrotic change; 2 for moderate collagen deposition and continuous fibrosis of the alveolar septa; 3 for excessive collagen deposition, thickened alveolar septa, compressed alveoli, and fibroplasia with confluent fibrotic mass.

### 4.5. Immunohistochemical Evaluation

Lung sections were incubated with anti-PARP antibody (1:250, Santa Cruz Biotechnology), as previously described by Cordaro et al. [50,51]. At the end of the protocol, the digital photos were analyzed by two observers blinded to the treatment, as previously performed in our laboratory [46,52,53].

### 4.6. Evaluation of Tissue Lipid Peroxidation

Malonaldehyde (MDA) levels were assessed, as previously described for lung tissue, at the end of the experiments. Briefly, after homogenization, MDA absorbance was measured at 650 nm using a spectrophotometer and expressed in mill-units per 100 milligram weight (mU/100 mg) of wet tissue [43,54].

### 4.7. Assessment of the Antioxidant System

SOD activity assay was performed following the method described by Marklund and Marklund at 420 nm [9,10,55,56]. The results are presented as SOD units/mg protein. Moreover, CAT activity was assessed following the method of Aebi in terms of mmoles of hydrogen peroxide (H_2_O_2_) consumed per min per mg of protein [57,58,59]. The results are presented as CAT units/mg protein. Furthermore, the level of the nonenzymatic cellular antioxidant glutathione was measured following the method of Moron et al. [60,61,62,63]. The results are presented as GSH units/mg protein.

### 4.8. Assessment of Cytokine Production

ELISA kits were used to investigate IL-1β, IL-18, IL-6, IL-10, and IL-4 in bronchoalveolar lavage fluid (BALF) at the end of experiment, as previously described [32,34,41,53,64,65,66,67]. The manufacturer’s manuals provided specific instructions for the test procedure.

### 4.9. Terminal Deoxynucleotidyl Nick-End Labeling (TUNEL) Assay

TUNEL staining for apoptotic cell nuclei and DAPI staining for all cell nuclei were performed in lung sections as described previously [34,55,68,69,70,71]. The index of apoptosis is expressed as the number of positively stained apoptotic cells over the total number of cells counted, multiplied by 100%.

### 4.10. Real-Time Quantitative Polymerase Chain Reaction Analysis (RT qPCR)

The levels of relative genes from lung tissues were measured by real-time quantitative polymerase chain reaction analysis (RT qPCR) using kits according to the manufacturer’s protocol (Aidlab Biotech, Beijing, China). The following primers were used: IL1β (F) ATGAAAGACGGCACACCCAC (R) AAGGCAGAGTCTTCGGTGAG; IL-6 (F) CAACCAAGAGGTGAGTGCTTC (R) GGTGTCCTCTTTCCCACACTG; IL-18 (F) GGAAGACCAGAGACATCCACTG (R) ACACTAGACCAAAGGGCTTG; IL-4 (F) CTGTAGGGCTTCCAAGGTGC (R) CTCTCATTGTGCCAGGTCACT; IL-10 (F) GCCAGTTAGAAAGCCACCAC (R) GGTTCAGCCTGTTTCCCAAC; ACTIN (F) GGCTGTATTCCCCTCCATCG (R) CCAGTTGGTAACAATGCCATGT. The expression levels of target mRNA were normalized to that of actin [64].

### 4.11. Materials

Vinclozolin (Lot #BCBZ5052) was obtained from Sigma-Aldrich INC. P.O. (St. Louis, MO, 63178, USA). Unless otherwise stated, all compounds were purchased from Sigma-Aldrich (Milan, Italy). All solutions were prepared using nonpyrogenic saline (0.9% NaCl; Baxter Healthcare Ltd., Thetford, Norfolk, UK).

### 4.12. Statistical Evaluation

In this study, all data are expressed as the average ± SEM and represent at least 3 experiments carried out on different days. For in vivo studies, N represents the number of animals used. The number of animals used for in vivo studies was determined using G*Power 3.1 software (Die Heinrich-Heine-Universität Düsseldorf, Düsseldorf, Germany). In all statistical studies, GraphPad Prism 8 software (La Jolla, CA, USA) was used. The results were analyzed by t-test, and a p-value less than 0.05 was considered significant.

## 5. Conclusions

In conclusion, in our study, we demonstrated for the first time that exposure to the fungicide vinclozolin, especially when chronic, induces serious alteration to the respiratory system, with an increase in inflammation, oxidative stress, and apoptosis. This is the first study to consider vinclozolin not only as an endocrine disruptor with androgenic activity but also as a toxin that will require a series of tests and regulations in order to evaluate its risks and hazards on the environment and animals.

## Data Availability

The data used to support the findings of this study are available from the corresponding author upon request.

## Supplementary Materials

The following supporting information can be downloaded at: https://www.mdpi.com/article/10.3390/ijms231911320/s1.
