# Supplementary material for: Chronic Exposure to Endocrine Disruptor Vinclozolin Leads to Lung Damage via Nrf2–Nf-kb Pathway Alterations
Source: Int J Mol Sci. 2022 Sep 26;23(19):11320. doi: 10.3390/ijms231911320 (PMC9569619; doi:10.3390/ijms231911320)
Supplement: Supplementary file 1 [file ijms-23-11320-s001.zip › ijms-1898858-supplementary.pdf]

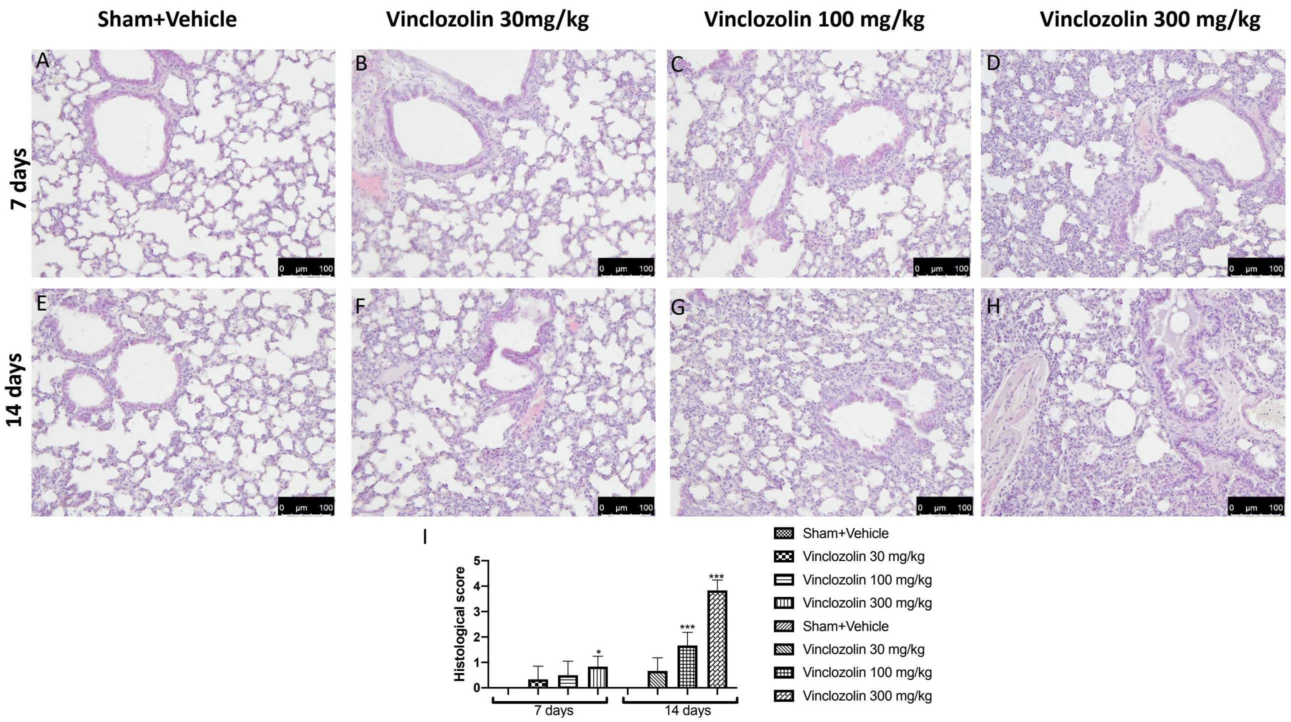

Supplementary Figure S1. Histological analysis of short term exposure of Vinclozolin at different concentration. 7 days for (A) Sham; (B) Vinclozolin 30 mg/kg; (C) Vinclozolin 100 mg/kg; (D) Vinclozolin 300 mg/kg; 14 days for (E) Sham; (F) Vinclozolin 30 mg/kg; (G) Vinclozolin 100 mg/kg; (H) Vinclozolin 300 mg/kg; (I) Histological Score . Values are means ± SEM of 6 mice for all group. Images shown is representative of the results obtained. See manuscript for further details. *** $p < 0.001$ vs. sham.
